# A review of limitations and potentials of desalination as a sustainable source of water

**DOI:** 10.1007/s11356-023-30662-x

**Published:** 2023-11-08

**Authors:** Babak Zolghadr-Asli, Neil McIntyre, Slobodan Djordjevic, Raziyeh Farmani, Liliana Pagliero, Victoriano Martínez-Alvarez, José F. Maestre-Valero

**Affiliations:** 1 Sustainable Minerals Institute, University of Queensland, Brisbane, Australia; 2 Centre for Water Systems, University of Exeter, Exeter, UK; 3 Agricultural Engineering Center, Technical University of Cartagena, Cartagena, Spain

**Keywords:** Desalination, Irrigation, Water resources management, Water crisis, Water-energy-food nexus

## Abstract

For centuries, desalination, in one way or another, has helped alleviate water scarcity. Over time, desalination has gone through an evolutionary process influenced largely by available contemporary technology. This improvement, for the most part, was reflected in the energy efficiency and, in turn, in terms of the cost-effectiveness of this practice. Thanks to such advancements, by the 1960s, the desalination industry experienced notable exponential growth, becoming a formidable option to supplement conventional water resources with a reliable non-conventional resource. That said, often, there are pressing associated issues, most notably environmental, socioeconomic, health, and relatively recently, agronomic concerns. Such reservations raise the question of whether desalination is indeed a sustainable solution to current water supply problems. This is exceptionally important to understand in light of the looming water and food crises. This paper, thus, tends to review these potential issues from the sustainability perspective. It is concluded that the aforementioned issues are indeed major concerns, but they can be mitigated by actions that consider the local context. These may be either prophylactic, proactive measures that require careful planning to tailor the situation to best fit a given region or reactive measures such as incorporating pre- (e.g., removing particles, debris, microorganisms, suspended solids, and silt from the intake water prior to the desalination process) and post-treatments (e.g., reintroducing calcium and magnesium ions to water to enhance its quality for irrigation purposes) to target specific shortcomings of desalination.

## Introduction

Undoubtedly, the spatial and temporal availability of freshwater resources is one of the primary challenges of water resources management, which is in turn one of the main pillars of achieving sustainable development on a global scale (Carlsen and Bruggemann 2022). At any given time, the available freshwater in a specific location is limited. On the other hand, global water demand has been increasing steadily, creating water stress in regions where the demand for water exceeds water availability (Hussain et al. 2022). While there is a large and considerable margin of uncertainty when one attempts to quantify these resources on a global scale, the most reliable recent estimates clearly indicate that there is a steady upward trend in the global average freshwater withdrawn, which is projected to continue into the foreseeable future (Ritchie and Roser 2017). Africa and Asia, for instance, are reported to have the lowest per capita freshwater renewal rates (UNESCO 2020). In addition to the spatial variably, temporal fluctuations can have major impacts on water availability. Often, the peak demand for water by different sectors, including and perhaps most notably the irrigated agriculture, does not coincide temporally with peak water availability, which often results in dry conditions. This creates additional water resources problems in semi-arid to arid regions such as the Middle East or some regions in southern Europe, which have notorious water scarcity issues (Bozorg-Haddad et al. 2020).

**Fig. 1:**
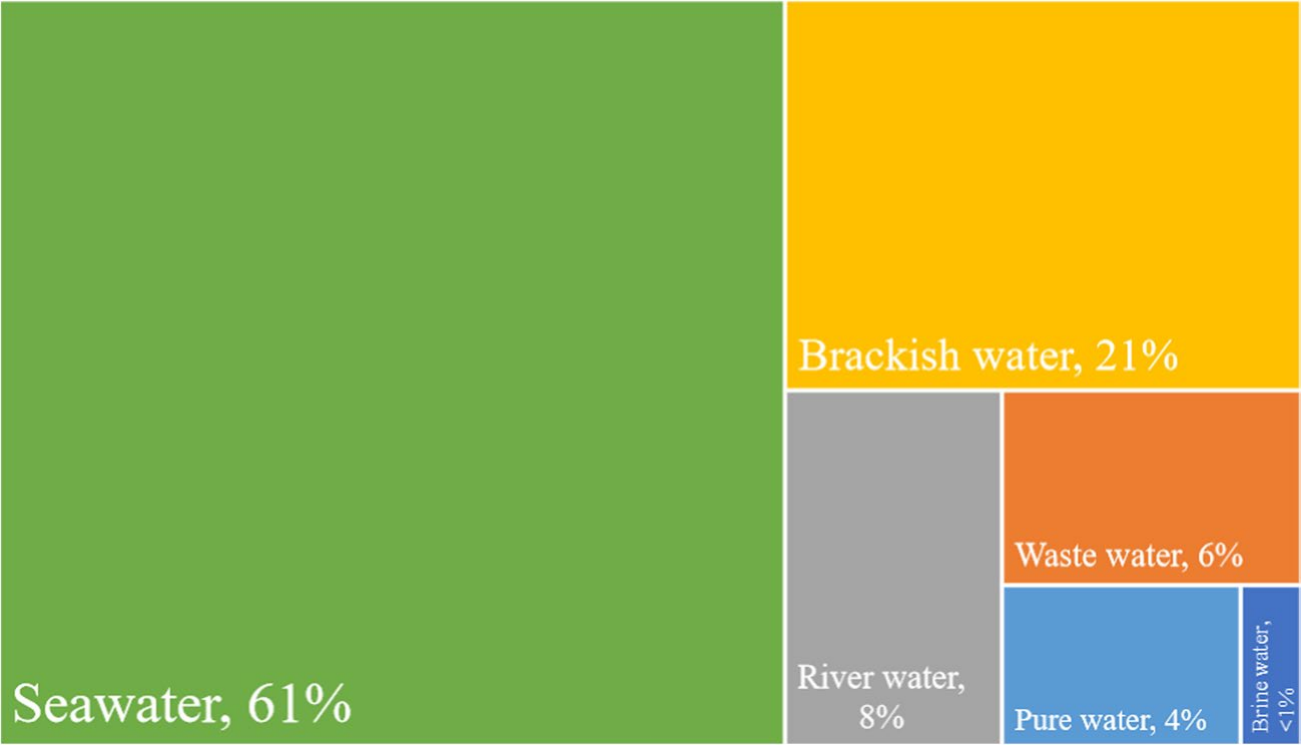
Various feed water sources were used in the desalination industry in 2018 (based on Saleh et al. 2019)

Desalination has always been perceived as a relatively reliable alternative to alleviate water crises. In short, the core idea behind desalination is to separate dissolved minerals from saline water, hence creating pure water. Perhaps one of the prominent features of desalination is its potential capacity to circumvent the barriers imposed by the hydrological cycle (Martínez-Alvarez et al. 2017). The idea is that, so long as steady saline feedwater is available, theoretically, the desalination units can be operated without notable influence from spatiotemporal conditions of the hydrologic cycle. The type of source selected to provide feedwater depends on the hydroclimatic and environmental context. Figure 1 depicts the feedwater source types used globally in the desalination industry in 2018 and shows that seawater desalination (61%), followed by brackish surface water or groundwater (21%), is the most significant sources of feedwater in the desalination industry.

**Fig. 2:**
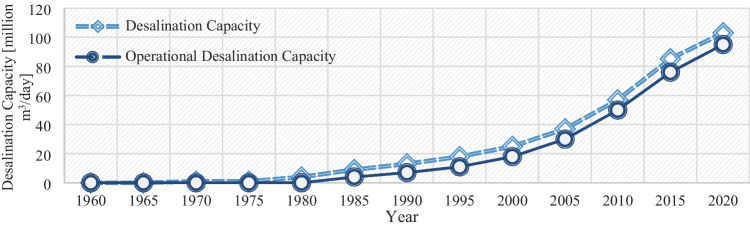
The evolution of the desalination industry across the globe over the years (based on Angelakis et al. 2021)

**Fig. 3:**
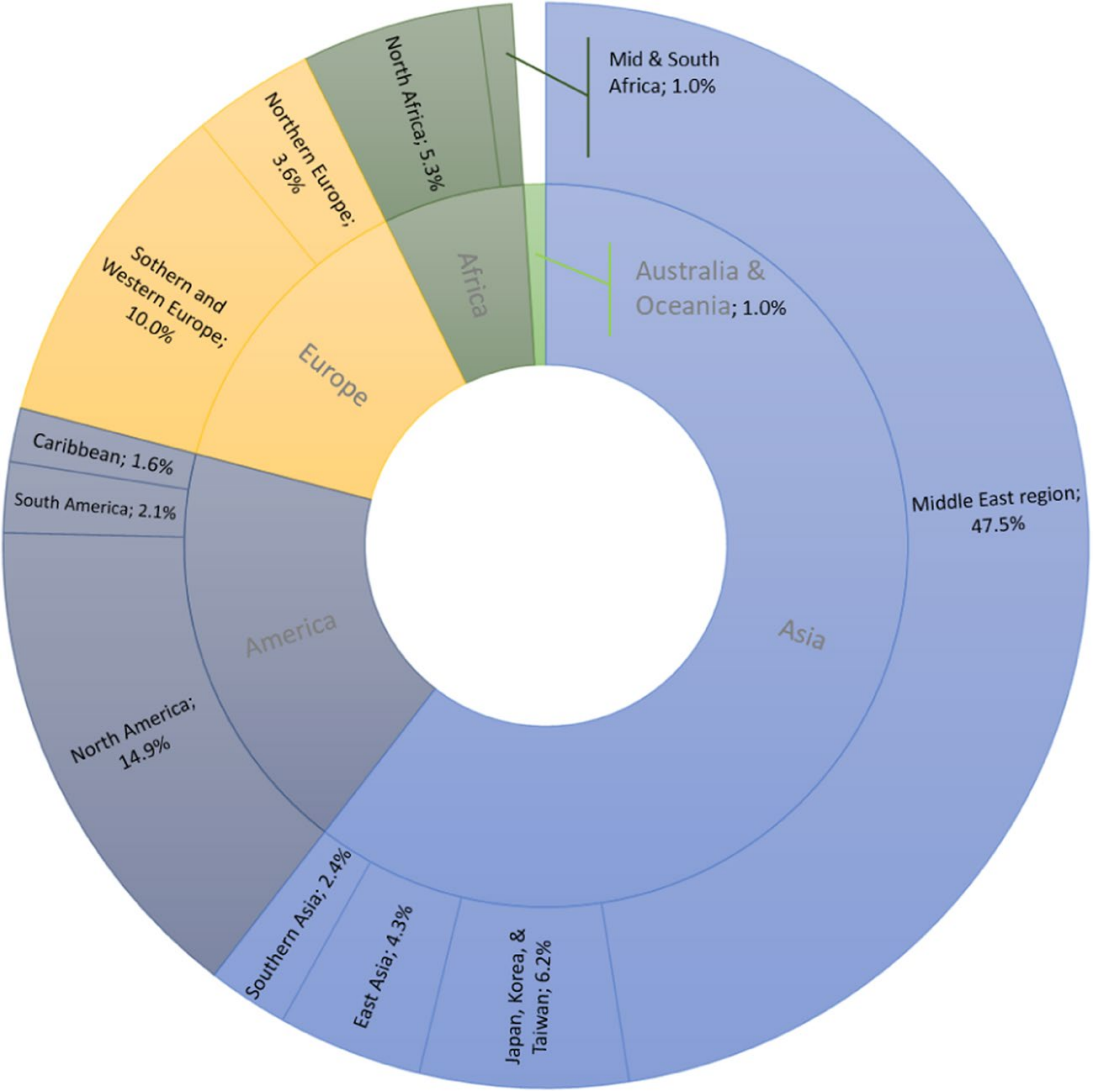
Relative regional share in available capacity for desalination (based on Lattemann et al. 2010)

Like many industries, desalination has had its fair share of ups and downs. For instance, this industry experienced a brief setback in the mid-2000s, primarily attributed to the market’s reaction to oil prices. Due to this new development in the market, some Middle Eastern countries have temporarily halted pursuing new desalination projects (Kucera 2019). That said, the overall projections for this industry have grown firmly since the 1960s across the globe (Aznar-Sánchez et al. 2017; Leon et al. 2021). Figure 2 shows how the desalination industry has evolved over the sixty years. All in all, it is fair to say that desalination has solidified its position as a formidable complementary source in today’s water resources landscape. That said, the degree to which desalination is practiced as a supplement to conventional water resources varies from region to region. Figure 3 depicts the global distribution of desalination capacity. As can be seen, the Middle East region alone accounts for 47.5% of global capacity.

As stated earlier, desalination could potentially be the solution to the looming water crisis, most notably in semi-arid and arid regions. However, what makes or breaks an idea, technology, or strategy, in general, is its capacity to maintain a sustainable performance. In this context, sustainability can be interpreted as maintaining a high-performance level in the long run without causing any significant adverse impact. As a potential remedy to water resources problems, the practice of desalination should also follow this principle. In that spirit, this review analyzes the applicability, merits, and potential drawbacks of desalination from a sustainability perspective and reviews the potential for desalination to accelerate its role in solving water resource challenges in water-stressed regions. The novelty of this approach is that it provides a more holistic viewpoint on how desalination can be used in a sustainable manner. In this context, sustainability pertains to acknowledging the multifaceted nature of water resources planning and management. In that spirit, the central premise of this research is to evaluate the practice of using desalinated water from different angles, most notably the environmental and socioeconomic implications associated with this practice, to unveil how and to what extent this technology could be incorporated into the water planning and management scheme of semi-arid and arid regions. The focus on sustainability complements other review papers that focus more on the technological (e.g., Subramani and Jacangelo 2015; Curto et al. 2021) or economic (e.g., Karagiannis and Soldatos 2008) sides of desalination. The covered topics are as follows: (I) a general outlook on the progression of desalination technology over time; (II) an overview of the known or potential environmental concerns concerning this practice; and (III) a critical analysis of the social acceptability of desalination.

## How has desalination technology evolved and adapted?

**Fig. 4:**
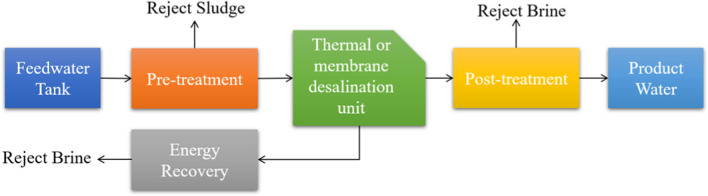
A simplified process diagram of thermal or membrane desalination units

The advent of the modern desalination era started with thermal desalination technology. The core idea behind thermal desalination is relatively simple; introducing thermal energy to the saline water would eventually turn it from a liquid to a gas phase (e.g., the vapor or steam) which would be considered free of salt, minerals, or other contaminants initially dissolved or suspended in the saline water. The vapor or steam would then be condensed back to the liquid form passed by desalination units. From a practical standpoint, there are different ways to implement the idea of thermal desalination, the most notable of which are multi-effect desalination (MED), multistage flash (MSF) desalination, and vapor compression (VC) (e.g., mechanical vapor compression (MVC) and thermal vapor compression (TVC)). A simplified process diagram of thermal desalination is depicted in Fig. 4.

**Fig. 5:**
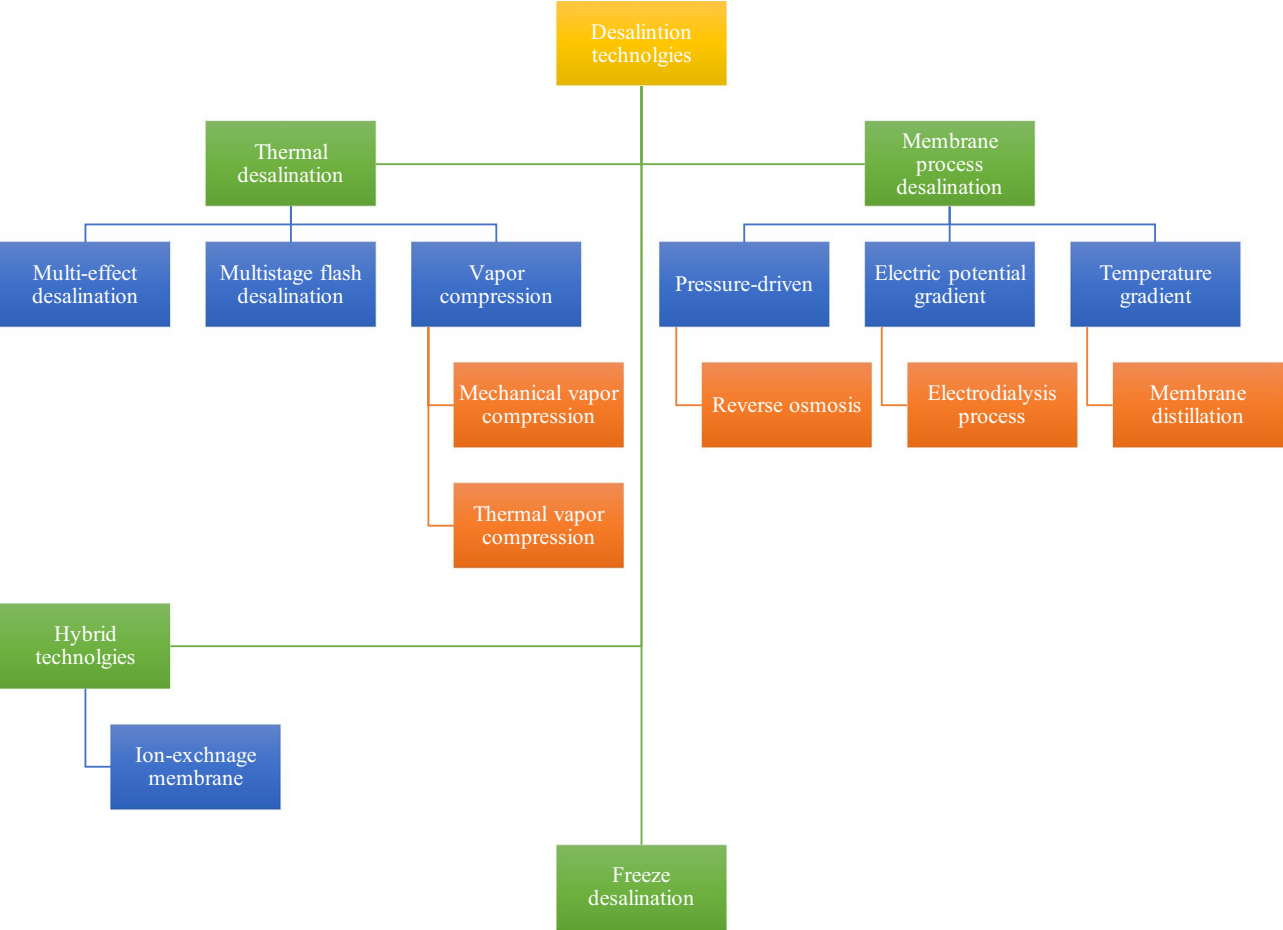
A schematic classification of the most promising desalination technologies

Another practical approach to desalination is through a phenomenon technically referred to as the membrane process. The central principle behind the membrane process is to enforce the transport of a substance, say water, through semi-permeable membranes against the chemical potential gradient. This process would allow the selective passage of particles through the membrane. In the context of desalination, to this day, there are three known practical ways to enforce the membrane process, namely, (I) pressure-driven, (II) using an electric potential gradient, and (III) using temperature gradient. A simplified process diagram of membrane desalination is depicted in Fig. 4. One of the most prevalent membrane process-based desalination units is reverse osmosis (RO). Other notable membrane process-based desalination are electrodialysis (ED), membrane distillation (MD), and freeze desalination (FD). It should be noted that forward osmosis (FO), hydrate-based desalination (HyDesal), and pervaporation desalination (PVD) are some of the other promising cutting-edge technologies that are for the most still in the research/development phase (Wang et al. 2016; Babu et al. 2018; Suwaileh et al. 2020). These and other well-known desalination technologies are categorized in Fig. 5.

**Fig. 6:**
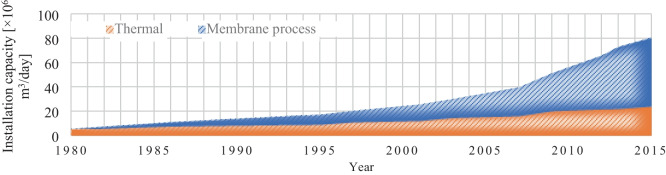
Cumulative installation capacity for thermal and membrane desalination technology from 1980 to 2015 (based on Kucera 2019)

**Fig. 7:**
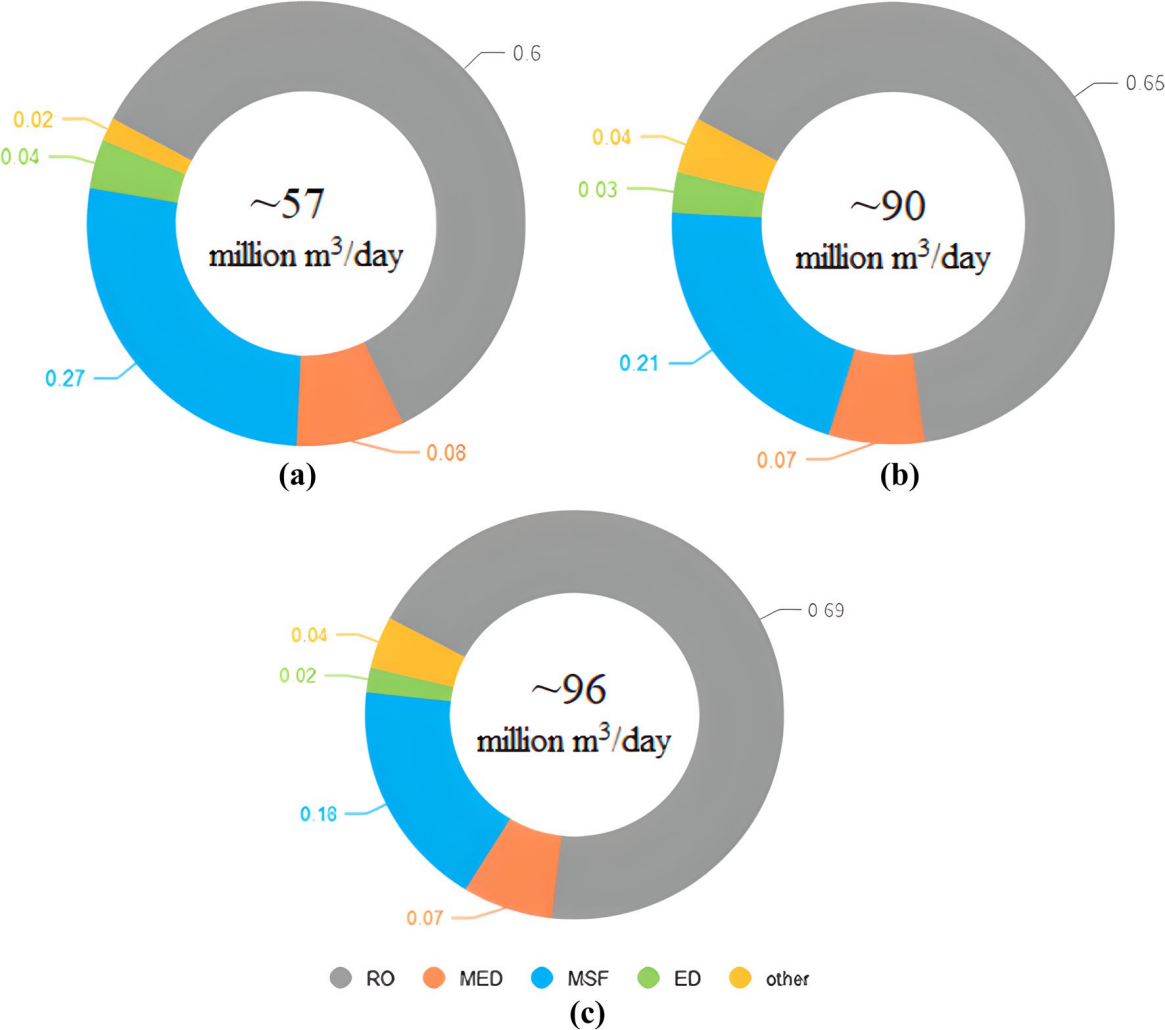
The relative share of each desalination technology in the global overall installation capacity in **a** 2010; **b** 2016; and **c** 2018 (based on Baten and Stummeyer 2013; Kucera 2019; Saleh et al. 2019; Angelakis et al. 2021)

In order to understand the current landscape of desalination in terms of the technologies being used, one must first appreciate how things have progressed over the years. Figure 6 depicts how two significant desalination classes, namely, thermal and membrane process, have evolved over the years. As seen here, while each technology’s installed capacity shows an upward trend, the pace of growth in the membrane process is more pronounced. What is important to take from this is that the data suggest that the growth of membrane process-based desalination accelerated around the mid-2000s. In fact, reportedly, 93% of newly installed desalination capacity from 2015 to 2016 on a global scale was based on some form of membrane process technology (Kucera 2019). This pattern can be further explored in Fig. 7, where the relative share of each major technology is dissected in 2010, 2016, and 2018. As can be seen, RO is the dominating technology in all three years, accounting for 60%, 65%, and 69% of the global installed capacity, respectively. MSF and MED technologies are second and third in terms of global capacity. While both these technologies are losing their share in the market, this is more pronounced in the case of MSF. Other technologies such as ED seem to have a small and steady share of the global installed capacity.

**Fig. 8:**
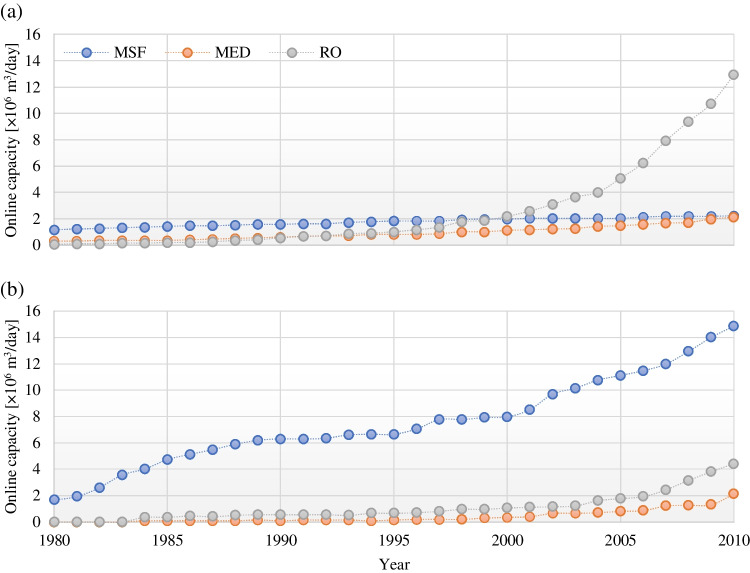
Cumulative online desalination capacity of **a** non-GCC and **b** GCC countries from 1980 to 2010 (based on Baten and Stummeyer 2013)

Figures 6 and 7, however, do not unveil all the current patterns in desalination technologies, as implementation varies from one region to another. For instance, analyzing the data from the cooperation council for the Arab states of the Persian Gulf (GCC), which consists of the United Arab Emirates (UAE), Bahrain, Saudi Arabia, Oman, Qatar, and Kuwait, reveals another side to this trend. Figure 8 depicts how GCC and non-GCC cumulative online desalination capacities have evolved from 1980 to 2010. Notable in this graph is the pace of growth in the desalination industry in GCC countries. Given that these primarily consist of nations with severe water challenges, the said trend is expected. However, while RO can be identified as the fastest growing desalination technology in non-GCC countries, MSF is, by a considerable margin, the most prevalent desalination technology in the GCC countries. A closer look at the data would reveal that from the early 2000s, the growth in RO technology in non-GCC countries accelerated, while in GCC countries, MSF technology boomed. In fact, thanks to the rapid economic growth during the oil boom of the 1970s, MSF technology has established its role in GCC nations, allowing them to cope with the rapid population growth of the said era (Baten and Stummeyer 2013).

As to why GCC nations seemingly favor MSF over RO technology, one must first note that, during the desalination boom in the said region, thermal technology was the only option with a proven track record, especially for large-scale plants (Baten and Stummeyer 2013). Easy access to cheap fossil fuels was another incentive that would justify resorting to thermal technologies (Baten and Stummeyer 2013; Kucera 2019). Finally, one should also take into consideration that the poor seawater quality of the region (relatively high salinity and temperature) for long was not compatible with membrane processing desalination units because it would cause biofouling of the membranes (Kucera 2019). It is worth noting that while the increase in salinity and temperature in feedwater could potentially decrease the efficiency of membrane desalination, they do not affect or favor thermal evaporation desalination (Altmann et al. 2022). These facts are reflected in the data in Fig. 8 and in a recent list of countries most reliant on thermal desalination—the United Arab Emirates (UAE), Saudi Arabia, Kuwait, Qatar, and Libya (Kucera 2019). However, modern membrane materials with higher permeability and salt-rejecting membranes that operate at lower pressure, as well as options for energy recovery and hybrid MSF-RO technology, have allowed RO to become a more viable, cost-effective option in the GCC region. Recent data on the use of RO in Saudi Arabia and UAE reflects this (Kucera 2019). As a result, currently, thermal desalination is almost exclusively used in installations that intend to use a waste heat source, such as thermal and nuclear power plants.

As stated, the current trends in the global desalination market show that RO is the most popular available technology (Figs. 6 and 7). In fact, countries such as Spain, Australia, and Algeria are investing heavily in RO for their national desalination programs (Kucera 2019). Various reasons can explain the popularity of this technology over traditional thermal desalination units, one being the lower capital costs required due to less-expensive construction materials and smaller infrastructure (Kucera 2019). In addition, while the operational costs of RO desalination units vary from case-to-case, they are often far less than MSF units and marginally better than MED units (Baten and Stummeyer 2013). In terms of environmental impacts and carbon footprint of these technologies, while it is important to note that this should ideally be determined on a case-by-case base, considering global averages, RO technology [2.562 (kgCO_2_*eq*/m^3^)] is a better choice than MSF [2.988 kgCO_2_*eq*/m^3^] and often competitive with MED units [1.280 kgCO_2_*eq*/m^3^] (Saleh et al. 2019). What is important to note here, however, is that the efficiency of RO desalination units can be improved through using energy-recovery systems and regular maintenance of the system (e.g., changing the RO membranes) (Dolnicar and Schäfer 2009; Cornejo et al. 2014; Leon et al. 2021). As a rule of thumb, the larger the desalination units, the more efficient the process becomes (Martínez-Alvarez et al. 2019). Using this fact within a water resources planning perspective could in some cases help offset the capital and investment costs.

The above discussion demonstrates that technological adaptations have made desalination more enticing and proficient, and this trend continues. However, the raw water quality and energy supply contexts have been critical drivers of technological solutions, and so appropriate solutions must be viewed on a regional and sometimes case-by-case basis.

## Environmental concerns about desalination

Several reports highlight significant concerns regarding the environmental impacts of desalination (McEvoy and Wilder 2012). This section reviews some of the most pressing environmental concerns cited in the literature and explores some potential remedies.

Some of the most pressing environmental issues are associated with water intake and discharge sites. The problem here is that in both situations, the desalination problem is innately interfering with the environment and ecosystem and, as such, can potentially disturb the established natural order. For seawater desalination, the intake problem, for instance, usually manifests itself as impingement and entrainment. The former refers to the losses of aquatic organisms as they collide with intake screens, while the latter describes the situation in which these organisms are drawn into the plant with the feedwater (Lattemann et al. 2010). It should be noted that the mere construction of intake infrastructures can cause disturbance in aquatic ecosystems and potentially endanger marine life. Infiltration galleries and beach-well intakes have been proposed as alternatives to open seawater intakes to mitigate such impacts (Lattemann et al. 2010).

Another inherent challenge in desalination is the effective disposal of liquid waste via discharge points. Based on the quality of intake water and the technology used in the desalination unit, the said by-product can be in the form of brine water or sludge. Brine water, which is concentrated seawater, can be seen in thermal and membrane-based desalination units. The second form of waste flow, however, has much lesser volume and importance due to the washing of filters (e.g., organic matter and suspended solids), and the chemical cleaning of the installations (e.g., detergents, acids, alkalis, antiscalant, antifouling agents) is primarily seen in RO units.

Traditionally, the produced sludge would have been mixed with brine. Reportedly, for seawater desalination, the sludge problem in the medium to large RO units (> 100,000 m^3^/day) that are prone to this specific challenge can even sum up to a few dozen tons per day (Baten and Stummeyer 2013). The current trend is, however, to incorporate installations in the desalination plants for the treatment and purification of these flows, and often thanks to proper environmental regulations, sludge disposal is no unmanageable issue in most cases. Apart from sheer volume, which can be problematic in and of itself, the chemical composition of the sludge can cause issues as well, most notably when the treated sludge is released directly back into the environment. In such cases, ferric salt may be employed in pretreatment (e.g., the process of removing particles, debris, microorganisms, suspended solids, and silt from the intake water prior to the desalination process) to remove the suspended materials. Though in and of itself, the presence of ferric salt may not cause a notable environmental impact, the discoloration caused by ferric salt can have an adverse aesthetic effect on the receiving water. This was, for instance, the case in Ashkelon, Israel (Baten and Stummeyer 2013). Alternatively, the pretreatment process can use centrifuge filtration that creates a high-solid waste product that can be disposed of in designated landfills. This, however, can be logistically challenging and introduce additional costs to the desalination process. As such, there is no universally accepted sustainable sludge disposal procedure, and for the most part, such strategies need to be determined on a case-by-case basis.

Disposing of brine is another common issue in the desalination industry in general. For instance, studies suggest that taking a global average, using RO technology, 1.5 m^3^ of brine is produced per every 1 m^3^ of desalinated water (Jones et al. 2019). Accordingly, improper brine disposal can lead to several environmental problems, ultimately endangering marine ecosystems like seagrass meadows or benthic populations (Pistocchi et al. 2020). However, the severity of these issues varies from case to case, to the point that under certain circumstances, it can even be considered negligible (Lattemann et al. 2010; McEvoy and Wilder 2012). This highlights the sheer importance of conducting an environmental impact analysis to get a better and more realistic sense of the potential impact of desalination on a local scale, as well as to develop environmental monitoring programs for the seafloor in the raw water intake and the brine discharge areas.

Like the sludge problem, brine’s chemical content, which depends on the desalination process and the feedwater quality, can also cause environmental concerns. Depending on these factors, brine composition could contain residual chlorine and chlorination by-products, antiscalant and antifoaming agents, and even certain heavy metals such as copper or nickel (Lattemann et al. 2010). The concentration of these chemical compounds is diluted following discharge, markedly if the discharge velocity is high. This, however, can result in the formation of high-salinity density currents that propagate hundreds to thousands of meters along the seafloor with limited dilution (Pistocchi et al. 2020).

Elevating seawater temperature near the discharging zones is another issue especially for thermal desalination (Kucera 2019). In MED, MSF, and VC desalination units, for instance, while there are certain preventive measures to tamper with the discharged brines, it often has higher temperatures than the seawater. The difference between the temperature and of the discharge and the ambient water dictates how these interact and mix. The chemical composition, due to its effect on buoyancy, also has an effect. Discharged water with negatively buoyant properties enhances the risk of plume sinking and seafloor spreading, endangering benthic ecosystems. Neutrally or positively buoyant, on the other hand, could cause plumes to spread in the water column and affect nektonic species (Lattemann et al. 2010; Kucera 2019). Though much remains unclear about the local environmental impacts of the discharge, to the point that such effects need to be determined on a case-by-case basis, often it is assumed that the design of outfalls, particularly outlet water velocity or mixing with cooling water from power plants, could mitigate or even negate such potential problems (Pistocchi et al. 2020).

Another pressing issue associated with desalination is greenhouse gas (GHG) emissions and, in turn, their potential contribution to climate change. Desalination is an energy-intensive technology, which implies that relying on this resource rather than conventional water resources would emit more GHG into the atmosphere (Martin-Gorriz et al. 2021). As such, it is believed that desalination can exacerbate climate change. It is also important to note that climate change adversely impacts marine ecosystems (Manes et al. 2021), making them more susceptible to potential risks associated with desalination technologies. Further in-depth studies are needed to quantify the nature and significance of these effects. Generally speaking, however, incorporating renewable energy resources as a substitute for conventional fossil fuels is a logical solution to the GHG emissions associated with desalination (Kucera 2019).

Utilizing desalinated water for irrigation could create additional environmental problems, most notably the potential for gradual degradation of soil structure (Dolnicar and Schäfer 2009). To understand the root of this problem, one should first note that the chemical composition of desalinated water often varies from other conventional water resources. Though the specifics may vary from one case to another, desalinated water is often expected to contain a higher concentration of Sodium and Boron ions (Silber et al. 2015; Martínez-Alvarez et al. 2019). Besides the well-documented phytotoxicity effect of the said element (Monterrey-Viña et al. 2020), prolonged exposure to excessive amounts of sodium could degrade the physical properties of the soil; specifically, it can cause clay dispersion (Martínez-Alvarez et al. 2016). The problem could manifest itself as deterioration of aggregate stability, increased susceptibility to surface sealing and soil erosion problems, soil compaction, and decreased soil aeration and hydraulic conductivity (Martínez-Alvarez et al. 2016, 2018). Concerning boron, although it is known to be an essential nutrient for fruits and vegetables implied in physiological processes, plant growth, and development (Hilal et al. 2011), its excess may represent a real boron-toxicity damage risk for sensible crops, and hence, different management options such as water blending or boron reduction strategies must be considered for the sake of sustainability (Imbernón-Mulero et al. 2022). Additionally, calcium and magnesium ions are almost completely removed in the desalination process; hence, reintroducing these elements through the post-treatment process may be a practical solution to mitigate the said effects (Martínez-Alvarez et al. 2018). Alternatively, this issue can be controlled through proper planning and management, such as adopting a suitable irrigation treatment to help leach the accumulated excess minerals (Martínez-Alvarez et al. 2018). However, improper execution of the latter plan could lead to washing the said elements into aquifers (Silber et al. 2015).

However, another argument could be made on the hidden environmental benefits of using desalination in the irrigated agriculture. The concept here is centered around the idea that this newly introduced resource would add water to the regional hydrological cycle (Pistocchi et al. 2020). Studies show that, on average, each cubic meter of plant transpiration yields 570 L of precipitation (Van der Ent et al. 2010). Implementing desalinated water for irrigation follows the same basic principle, although quantifying its local significance would require reconstruction of the relevant climate variables at high resolutions (Van der Ent et al. 2010). It should be noted that, however, given the general time and space scales of recycling of soil moisture in arid regions, in most cases, this seems likely to be an insignificant effect. The importance of this phenomenon notwithstanding, a more apparent positive effect of introducing desalinated water to meet agricultural demands is that it reduces the pressure on other water resources. Problems that would otherwise result from over-abstraction from freshwater resources could be mitigated to some extent (Calatrava et al. 2022).

All in all, all environmental issues associated with desalination seem to be manageable, and this practice could potentially have some environmental upside. As a final note in this section, it is essential to emphasize the importance of conducting environmental impact assessments on a case-by-case basis, as the magnitude of environmental impacts will vary based on the unique characteristics of a given region.

## Perceptions toward use of desalinated water and its socioeconomic implications

When one tries to analyze the practice of using desalinated water through the lens of socioeconomics, perhaps one of the most central, yet scanted, factors that determine the acceptability of this practice, in general, is the notion of *perception*. Perception can be seen as *one’s primary form of cognitive contact with the world around oneself* (Efron 1969), which governs how we generally lean toward a new concept or practice. From the sustainability standpoint, this notion can have profound implications when introducing an alternative and often unconventional source to meet consumers’ demands. The collective perception of consumers, stakeholders, and decision-makers toward the said resource can ultimately and inevitably shape the future role it plays in the broader context of water management on the local and perhaps global scale. In theory, while we can expect the positive perception associated with a practice or an idea to push the public toward accepting or embracing the newly introduced nuances, the stigma attached to these notions can, on the other hand, adversely affect the public acceptance and behavior patterns. With that idea in mind, this section touches on the idea of how perceptions about implementing desalinated water have formed and can shape the future of this practice.

The influence of the public’s perception toward the acceptance of an idea or a practice is, in effect, a double-sided blade, as in such opinionated collective behaviors of the society can also be targeted to promote or persuade the public concerning a given practice or a concept. For example, the socio-environmental movements highlighting the potential threats behind plastic pollution are cases where such movements have raised public awareness, showcasing the positive side of perception’s influence on public behavior (Soares et al. 2021). As a result of such movements, in recent years, communities have actively tried to address this issue by recycling their used plastics or incorporating more environmentally friendly alternatives to plastics materials within the production lines, even though often these solutions might not be the most economical or perhaps convenient way for the manufacturers or the consumers.

These examples demonstrate how, in a practical context, the public’s perception can contribute positively or negatively when it comes to adopting a new idea or practice within a given community. This begs the question, how and to what extent can such perceptions shape the general practice of using desalinated water.

It is reasonable to acknowledge that the economic costs of desalination might be highly significant from the standpoint of stakeholders and policymakers during a water crisis. Specifically, this pertains to the comprehensive costs associated with harnessing, conveying, and distributing such resources. These costs encompass both capital expenditure (CAPEX) required for establishing the necessary infrastructure and operational expenses (OPEX) necessary for the ongoing operation and management of this infrastructure. While the specific CAPEX and OPEX figures vary substantially in case-by-case basis (Karagiannis and Soldatos 2008), they consistently tend to exceed the costs associated with most conventional resources. To address this, the introduction of tariffs, for instance, has been employed as an economic lever to render these resources a more economically viable option (Pinto and Marques 2017a, b). It is important to note, however, that the decision to utilize such resources in semi-arid and arid regions is not solely driven by economic considerations, as mentioned earlier. On that note, as will be explored in greater detail later on, while water pricing remains a contributing factor, it alone cannot determine the socioeconomic acceptability of water resources. In light of this, it becomes crucial to comprehend the multifaceted factors that influence stakeholders’ perceptions regarding these resources.

A systematic review on this subject would quickly unveil that this specific topic has received little to no attention from scholars and researchers in the past. Only a handful of papers tackled the notion of perception regarding implementing desalinated water (e.g., Dolnicar and Schäfer 2009; Dolnicar and Hurlimann 2010; Fielding et al. 2018; Etale et al. 2020). Overall, based on extensive research and exhaustive data gathered from all around the globe on this topic, it is safe to conclude that often, people have a somewhat negative perception of using desalinated and recycled water for urban use, especially when it comes to human consumption and bathing (Fielding et al. 2018). That said, the extent of this reluctance varies from one case to another. More relevantly, studies show that this negative stigma is presumed to be less pronounced when it comes to using desalinated water in comparison to the other options that are based on recycled and reclaimed water (Etale et al. 2020).

According to studies, when it comes to using desalinated water for urban use, the central pillars that shape this negative persona from a public perspective, in no particular order, are health risks, cost, environmental concerns, disgusts, level of knowledge, and previous experiences with water shortages (Etale et al. 2020). Needless to say, it is suggested by these surveys that the degree to which these factors come into play varies case-by-case. For instance, feelings were so pronounced and intense in some cases that even financial compensation would not change or mitigate the situation. This was the case in a survey in Kuwait, where even bringing down the price for the customers would not convince them to implement these resources in their day-to-day uses (Dolnicar and Schäfer 2009).

That said, findings from these studies suggest that such perceptions can be manipulated through a series of external factors. These can be seen as mechanisms to persuade the public and reshape their opinions regarding the application of desalinated for urban use. Some of the most cited factors in no particular order are demographic factors (e.g., age, gender, education, occupation), level of trust in authorities and decision-makers, and cultural and religious concerns (Etale et al. 2020). That said, it should be noted that there are no universal recipes when it comes to convincing the public about alternative, unconventional water resources, and this is, more than anything, a case-by-case situation.

Understanding the full scope of this concept within the context of the irrigation and agriculture sector, however, is more complicated. A good showcase of this would be a survey that was conducted among irrigators in the southeastern part of Spain (Ricart et al. 2020), in which farmers in different communities were asked to rank their perceptions about the quality of desalinated water. Among the surveyed groups, only two gave this resource unfavorable scores. Interestingly, these are two neighboring communities, one of which did not even use desalinated water for irrigation when this survey was conducted. The said community even gave the desalinated water a lower score than the group that used this source for irrigation. As reported by Ricart et al. (2020), the water used by the neighboring community had contained some elements (i.e., Boron) that can be toxic for irrigation purposes, which resulted in their grading the desalinated water quality as “fair.” The communication of this gave the second community a bad and arguably unrealistic impression about the quality of desalinated water. This incident showcases how perception can influence the acceptability of this practice in real-world situations.

When it comes to understanding the role of perception in the implementation of desalinated water for irrigation, one of the crucial things that induces further complications to an already complex problem is the vast and diverse number of players involved. The primary stakeholders and funders of desalination projects represent another aspect that can introduce complexity into the equation. Historically, public sector and governmental agencies have been the primary backers of desalination units (March 2015). However, in recent times, many projects have been emerging under the purview of private entities or public–private consortiums (Greer et al. 2021). This shift in sponsorship brings forth distinct perspectives and priorities, potentially adding intricacy to the governance and regulation of such units. Consequently, it becomes more challenging to ascertain and dissect the perceptions of stakeholders regarding desalination in a broader sense (Campero and Harris 2019).

As for the main stakeholders’ viewpoints, in the case of urban uses, for instance, it could be argued that the public consumers would clearly dominate the role of perceptions. In agriculture, however, in addition to the direct consumer, the farmers and irrigators, the perception of the public, as the leading consumer of the agricultural products, is expected to play an important role. In addition to this, governing bodies, health and environmental agencies, regulators, and private investors are also, each to a certain degree, involved in the decision-making process. While in some of these cases, the objective assessment and scientific findings may eventually be the decisive factors, one cannot underestimate how the public’s perception, as shown in previous cases (e.g., Ricart et al. 2020), can ultimately be a contributing factor.

With these in mind, we can finally look at the limited number of papers that exclusively addressed this notion within the context of using desalinated water for irrigation (e.g., Aznar-Sánchez et al. 2017; Ghermandi and Minich 2017; Ricart et al. 2020; Villar-Navascués et al. 2020). The obtained results, however, varied vastly on a case-by-case basis. The idea here is that while there may be a general pattern behind the public’s perceptions, based on the findings of similar studies reviewed earlier in this section, it is safe to assume that different regions may react differently to this practice. In other words, while we expect to see recurring factors when studying the gist of the public’s perceptions toward irrigation with desalinated water, odds are there would be notable variations based on the unique circumstances of each given case. A comprehensive apprehension of such behaviors would depend on unveiling these very local-dependent contributing factors.

When it comes to farmers’ perceptions of the practice of using desalinated water for irrigation purposes, certain factors seem to be recurring in most case studies. These fundamental factors, in no particular order, are found to be the economic aspect of the operation and, more specifically, the cost of desalinated water, the quality and quantity of desalinated water, environmental concerns, an insurance mechanism to meet the contracts between the decision-makers and irrigator community to guarantee the availability of desalinated water under the promised circumstances, the quality of the agricultural products and soil, lack of any storage system for storing the excess desalinated water, and finally, contracts that are based on the take-or-pay paradigm (Aznar-Sánchez et al. 2017; Ghermandi and Minich 2017; Ricart et al. 2020; Villar-Navascués et al. 2020). Of course, how relevant these factors would be for a given region must be determined on a case-by-case basis.

As for the studies that focused on this research topic, three out of four papers mentioned earlier focused on Spain. The findings of these studies suggest that, overall, the farmers in this region generally have a somewhat negative perspective on the idea of using desalinated water for irrigation purposes (Aznar-Sánchez et al. 2017). This is to the point that in some cases, even if the exact same price applies to different water resources, the farmers tend not to opt for desalinated water in any way, shape, or form (Aznar-Sánchez et al. 2017). However, recent studies in the region have demonstrated that this negative perception is seemingly improving over time (Villar-Navascués et al. 2020).

In contrast to results obtained in Spain, farmers are reportedly keener about the application and the future of this practice in Israel. Based on research conducted in 2017, about half of the surveyed farmers were planning to switch to desalinated water in the next few years (Ghermandi and Minich 2017). The general conclusion of that study was that farming communities in Israel are open to the idea of using desalinated water as a reliable source for irrigation. That result, in and of itself, shows that there are crucial factors that need to be studied case-by-case. And, more than anything, it demonstrates the massive and tangible gap in knowledge that exists when it comes to understanding the irrigators’ perceptions toward desalinated water use in general.

That said, while two ostensibly contradictory opinions are reported above, a more general pattern would unveil itself if these perceptions were to be analyzed using a more holistic perspective that accounts for the water resources situation in these regions. Seemingly, the more water-stressed a region, such as the case in Israel, when the farmer community has not many alternative water resources at their disposal, the more favorable the views toward desalinated water use. In contrast, in a place such as Spain, where desalinated water is still viewed as a complimentary resource by farmers for use only when they cannot meet their demand through conventional water resources, these perceptions are not as favorable. What is important to note here is that such perceptions can potentially adjust to match the current water situation in a given region. Take the case of Spain’s 2005 drought, where the request for this type of resource, perhaps out of necessity, increased drastically (Martínez-Alvarez et al. 2019). A similar situation occurred during the Australian millennium drought between 2003 and 2012 (Heihsel et al. 2019). As such, it could be argued that, often, the imminent threat of drought, or water scarcity to be more general, can be enticing enough to convey the use of this unconventional resource from the farmers’ perspective.

## Concluding remarks

Although the earliest application of commercial desalination dates back to the late nineteenth and early twentieth centuries, it was not until the 1960s that this practice eventually evolved into a viable solution to combat water shortages in semi-arid and arid regions. While this practice has experienced exponential growth in recent years in water-scarce regions, there are still some unexplored angles regarding the potential impacts of desalination. For the most part, this is because it would take years for such effects, including environmental repercussions, to be detected with a scientific degree of certainty. As the desalination sector is anticipated to continue expanding globally, in accordance with the sustainable development principle, it is necessary to understand and, in turn, minimize these adverse effects. In fact, it could be argued that these would define the evolution of this industry in the foreseeable future. On that note, an excellent universal policy would be that desalination projects need to demonstrate sustainability on economic, environmental, and social grounds and demonstrate that the technologies and designs employed maximize sustainability for the particular local context of the project. Consequently, this review intends to take a more holistic approach to assess the general practice of desalination through the aforementioned lenses.

The results of this review show that from a practical point of view, environmental and socioeconomic concerns are some of the pressing issues concerning desalinated water. For the most part, the socioeconomic concerns with regard to desalination are centered around the overall cost of desalinated water, which, in turn, is rooted largely in the energy consumption of desalination units. As an energy-intensive technology, energy markets can profoundly impact how cost-effective this practice can be. However, the core technology behind desalination has improved immensely from where it started, to the point that state-of-the-art RO units are far more proficient in energy consumption than they used to be. RO units seem to have established their place as the standard practice in the market. Current global trends show how they are and most likely continue to dominate the desalination industry for the foreseeable future. In fact, even the Middle Eastern nations that long relied on previous-generation thermal technologies are gradually moving toward RO-based units. Switching from conventional fossil fuels to renewable resources could be another groundbreaking strategy to make these technologies more cost-effective and environmentally friendly.

As for the remaining issues concerning the environmental-related impacts, the findings of this review suggest that the expected effects can drastically vary from one case to another. This, more than anything, highlights the necessity of conducting region-based studies to unveil such impacts on a local level. That said, the gathered data suggests that all the said issues can be mitigated, often substantially. In some cases, well-thought planning and management measures provide a tailor-fitted solution to optimizing the efficiency of the desalination units. In other cases, a more hands-on measure, such as establishing pre- and post-treatments, could minimize the adverse effects of desalination.

On the other, there are some notable advantages to incorporating desalinated water in the grand scheme of water resources planning and management. First and foremost is the augmentation of a versatile and reliable resource that can, by nature, circumvent hydro-climatic oscillations. If executed correctly, this would facilitate an effective, sustainable water resources planning and management scheme. Doing so, however, requires addressing the fundamental question of how desalination, in general, should be utilized in the context of other available water resources. The point is that desalinated water can be seen as an augmentation source that comes into play mostly during peak demands. This is to say that along with other water management schemes, most notably demand management, introducing desalinated water in the proper context can help target sustainable development goals. On the other hand, desalinated water can also be treated as a means to meet the baseline demands, providing a unique socioeconomic opportunity. However, opting for the latter option requires careful planning and management. What ensures sustainability, in short, is how this technology can endure its cost-effective performance with minimum to no environmental degradation in the long run. This is, in turn, reflected in how effectively and quickly decision-makers can address the pressing issues. This becomes quite crucial, yet more challenging, as more sectors, including and perhaps most notably the agriculture industry, are gradually leaning toward desalinated water.

Finally, one cannot assess the sustainability of desalination without exploring its formidability from the public perspective. Our systematic review of this subject revealed that this specific topic has received little to no attention. That said, the limited available studies point out the presence of a potential negative stigma both for urban and irrigation use of desalination water, though the extent and reasons for these vary vastly from one case to another. That said, these studies also highlight that the public can be persuaded on this matter should the right and tailored mechanism are to be utilized in a timely and proper manner. What is also important to note here is that one can see that, at least in the context of irrigation demands, perceptions can potentially adjust to match the current water situation in a given region. The gathered data suggests the more water-stressed the region, the more the public tends to lean toward these unconventional resources.

## Data Availability

All used data have been presented in the paper.
